# Supplementary material for: Case Reports for Topical Treatment of Corneal Ulcers with a New Matrix Therapy Agent or RGTA® in Dogs
Source: Vet Sci. 2019 Dec 13;6(4):103. doi: 10.3390/vetsci6040103 (PMC6958328; doi:10.3390/vetsci6040103)
Supplement: Supplementary file 1 [file vetsci-06-00103-s001.pdf]

Table S1: Characteristics of the case population.

Dog#	ID#	Gender	Intact, Spayed or Neutered	BW (kg)
**1**	12285	F	S	11.0
**2**	13204	M	I	11.0
**3**	13256	M	N	10.8
**4**	13206	M	I	40.9
**5**	13201	F	S	4.0
**6**	13250	M	N	11.3
**7**	13266	M	N	10.8
**8**	13194	F	S	6.2
**9**	13241	M	N	5.9
**10**	12840	F	I	3.2
**11**	11031	F	S	4.4
